# Varroa Destructor Classification Using Legendre–Fourier Moments with Different Color Spaces

**DOI:** 10.3390/jimaging9070144

**Published:** 2023-07-14

**Authors:** Alicia Noriega-Escamilla, César J. Camacho-Bello, Rosa M. Ortega-Mendoza, José H. Arroyo-Núñez, Lucia Gutiérrez-Lazcano

**Affiliations:** Artificial Intelligence Laboratory, Universidad Politécnica de Tulancingo, Tulancingo 43629, Hidalgo, Mexico; alicia.noriega.1731021@upt.edu.mx (A.N.-E.); rosa.ortega@upt.edu.mx (R.M.O.-M.); humberto.arroyo@upt.edu.mx (J.H.A.-N.); lucia.gutierrez@upt.edu.mx (L.G.-L.)

**Keywords:** Legendre–Fourier multichannel moments, honey bee, Varroa destructor

## Abstract

Bees play a critical role in pollination and food production, so their preservation is essential, particularly highlighting the importance of detecting diseases in bees early. The Varroa destructor mite is the primary factor contributing to increased viral infections that can lead to hive mortality. This study presents an innovative method for identifying Varroa destructors in honey bees using multichannel Legendre–Fourier moments. The descriptors derived from this approach possess distinctive characteristics, such as rotation and scale invariance, and noise resistance, allowing the representation of digital images with minimal descriptors. This characteristic is advantageous when analyzing images of living organisms that are not in a static posture. The proposal evaluates the algorithm’s efficiency using different color models, and to enhance its capacity, a subdivision of the VarroaDataset is used. This enhancement allows the algorithm to process additional information about the color and shape of the bee’s legs, wings, eyes, and mouth. To demonstrate the advantages of our approach, we compare it with other deep learning methods, in semantic segmentation techniques, such as DeepLabV3, and object detection techniques, such as YOLOv5. The results suggest that our proposal offers a promising means for the early detection of the Varroa destructor mite, which could be an essential pillar in the preservation of bees and, therefore, in food production.

## 1. Introduction

The honey bee (Apis mellifera) is a species native to Africa, Europe, and western Asia; its management has contributed to the presence of this species in all continents except Antarctica and some oceanic islands [1]. They are vital as pollinators, visiting more than 90% of the world’s principal crops [2]. They also generate essential products such as honey, pollen, propolis, and royal jelly, producing jobs mainly in rural areas [3]. Hence, reducing their population could affect the production and quality of food whose crops depend on insect pollination [4]. Despite its relevance, a decline in bee diversity has been recorded due to climate change, pesticide use, and loss of natural habitats [5]. In addition, diseases associated with fungi, bacteria, viruses, and invertebrate parasites threaten the health of bees [2]. These diseases tend to spread to nearby bee populations due to commercial management, mass breeding, transport, trade, and physical contact between bees, especially during harvesting, representing a problem for conserving wild bee species.

The worldwide transmission and spread of the ectoparasitic mite Varroa destructor is the main factor in increasing viral infections. Furthermore, it inoculates in the larvae and adults, causing the death of hives [6]. A decrease in production of 45% is estimated in a swarm of domestic bees infested with Varroa, which causes economic losses due to sanitary treatments, the repopulation of the packs, the treatment of secondary diseases, and labor [7]. Currently, different chemicals and application methods keep the mite population under control. However, to achieve an optimal effect, these control methods must consider the climate, the conditions inside the hive, and the mode of application [6].

Nowadays, diagnostic tools are an essential component of integrated pest management. Monitoring the level of infestation requires washing, separating, and counting the number of bees and mites. Therefore, proposals have been made to evaluate and monitor different factors that allow the health of bee colonies to be tracked with minimal human interaction through computer vision systems. For example, Rodríguez et al. [8] perform pollen detection by analyzing the hive entrance, where they perform a comparative analysis with images of pollen-bearing and non-pollen-bearing bees using different types of neural networks. Sledevivc [9] presents image classification with pollen-bearing bees using a convolutional neural network. Marstaller et al. [10] propose DeepBees, which is a non-invasive system to monitor hives using computer vision based on a Raspberry Pi by transferring cropped image sequences of each insect for pollen detection and bee pose.

On the other hand, the work presented by Tiwari [11] uses deep learning to recognize bees in videos and monitor the behavior of the colonies through traffic analysis; this variable is essential to observe the availability and demand for food, the age structure of the colony, and the impact of pesticides. Rodríguez et al. [12] develop a system for detecting, locating, and tracking bee body parts from a video at the entrance ramp of the colony. Tashakkori et al. [13] implement a data collection and monitoring system called Beemon that captures sensor data (temperature, humidity, and weight) and sends video and audio recordings, as well as input to hives, for analysis and research.

Regarding pest detection, Bjerge et al. [14] describe a portable computer vision system that performs infection detection and automatic counting of bees to determine the level of infestation and minimize damage to the colony. This system uses a multispectral camera and illumination with blue, red, and infrared LEDs to facilitate Varroa separation using machine vision algorithms based on deep learning. Sevin et al. [15] develop the Var-Gor system using bee passage tunnels, an autofocus detection camera, and a solar-panel-generated power supply to detect Varroa mites. Bilik et al. [16] present an object-detector-based method for monitoring the health status of bee colonies with a dataset of 600 images of healthy and infested bees in various scenes, and the detectors reach 87% in the detection of infested bees and up to 71% in the detection of the Varroa destructor mite itself. Schurischuster and Kampel [17] classify bees into two classes, healthy and infested with the parasitic mite Varroa destructor, through images acquired at the hive’s entrance. They compare two classification methods based on AlexNet and ResNet and a semantic segmentation approach using DeepLabV3, with the latter achieving a classification of 90.8% with an F1 score of 95%.

The mentioned works use extensive databases because deep learning models require a large amount of information for their training and usually use data augmentation to improve their results [14,16]. In addition, these systems present problems for the correct classification of Varroa, such as the bee’s orientation at the time of acquiring the image and the characteristic color of Varroa being confused with parts of the bee. Therefore, new strategies are required to deal with this type of problem. In this context, circular moments are a novel tool to represent information with minimal redundancy and invariance to transformations of geometric type [18], which allows the classification of images with reduced databases without requiring data augmentation or image preprocessing.

Recently, the multichannel approach emerged as a new proposal to extract features in RGB images showing higher efficiency than the quaternion moments. Sing and Sing [19] present the orthogonal multichannel moments based on Zernike moments (ZMs). Similarly, Hosny and Darwish [20] present a new set of Chebyshev–Fourier multichannel moments (MMCFs), introducing new sets of multichannel moments [21,22,23,24,25]. Furthermore, the moments distinguish the characteristic color of objects in images with a reduced number of descriptors [19].

There is a large number of families of moments capable of characterizing images; however, studies have shown that Legendre–Fourier moments perform well with respect to other moments [26,27,28,29]. On the other hand, using different color models to analyze different image features is common. For example, Bolappa Gamage et al. [30] evaluate the nutritional status of strawberry crop leaves using the RGB color model; this same color model is also used by Malgorzata et al. [31] to analyze the health status of horses after exercise. Wan Yuanbin et al. [32] perform image segmentation in RGB and YCbCr color models for forest fire image recognition. The works presented to identify the Varroa mite describe problems differentiating between the mite and the bee’s eye due to the color similarity [16]. In addition, as they are living beings, it is difficult to keep the bee’s capture in a single position [33], so it requires descriptors robust to different changes.

In this research work, an alternative method is proposed to classify bee images using Legendre–Fourier multichannel moments. The descriptors used have relevant qualities that allow the representation of digital images with a minimum number of descriptors; this characteristic is beneficial in cases where images of living beings that do not keep a static position are analyzed. The multichannel moments provide the information of each channel of the color space necessary to carry out the classification with greater accuracy and a lower computational cost. The proposed algorithm is also used with different color models to evaluate its effectiveness.

It is essential to mention that deep learning neural-network-based methods such as semantic segmentation and object detection have shown high performance in numerous computer vision challenges. Although these methods are relevant and have demonstrated excellent performance, they have limitations, especially regarding computational demand and the need for large volumes of data. Our study tried to address these problems using Legendre–Fourier multichannel moments, providing an alternative for handling images of living beings in various positions with fewer descriptors and less computational cost. One of the innovative approaches in object detection is provided by Li et al. [34], which explores using single-vision transformers for object detection, demonstrating performance comparable to convolution networks. In addition, Wang et al. [35] present a new state-of-the-art in real-time object detector, further improving the precision and speed of object detection in images. Although these methods are highly effective, they rely on complex network structures and large amounts of training data, which can be challenging in resource-constrained environments or where the data volume is insufficient. Improving image quality can be crucial to effective object detection, as shown by Liu et al. [36]. This study uses an image enhancement guide to improve object detection in visually degraded scenes. However, this approach could be problematic since the image enhancement could alter the color characteristics critical for detecting the Varroa destructor. On the other hand, Su et al. [37] introduce an efficient method of detecting objects without anchors, which could be relevant to our study. However, this method still requires a lot of training data, which can be problematic. Regarding semantic segmentation, Strudel et al. [38] and Zhang et al. [39] present efficient transformers for semantic segmentation. These approaches achieve high performance in segmentation tasks, but as with the method mentioned above, these detection methods require large amounts of training data and significant computational resources. Finally, Yan et al. [40] introduce an approach to domain adaptation that could be relevant in our case if the training and test data come from different domains. However, this method may not be necessary if our training and test data are consistent regarding image quality and shooting conditions. Therefore, although these methods have shown significant performance in their respective fields, their limitations may make them less suitable for our Varroa destructor classification task. Based on Legendre–Fourier multichannel moments, the proposal seeks to address these limitations by providing an alternative that can handle images of living beings in various positions with fewer descriptors and less computational cost. The present approach can give comparable or even superior results to those obtained with deep-learning-based methods that classify Varroa destructor using semantic segmentation [17] and object detection [16], with the added advantages of lower data demand and computational cost.

The document is organized as follows. Section 2 briefly describes the database to be used, the definition of multichannel Legendre–Fourier moments, and the different test color spaces. Section 3 presents the strategy to improve the classification of the Varroa mite in bees. Section 4 shows the results and discusses this work’s advantages compared to current techniques. Finally, we provide the conclusions of the work carried out.

## 2. Materials and Methods

### 2.1. Database of Honey Bees with Varroa

**Figure 1:**
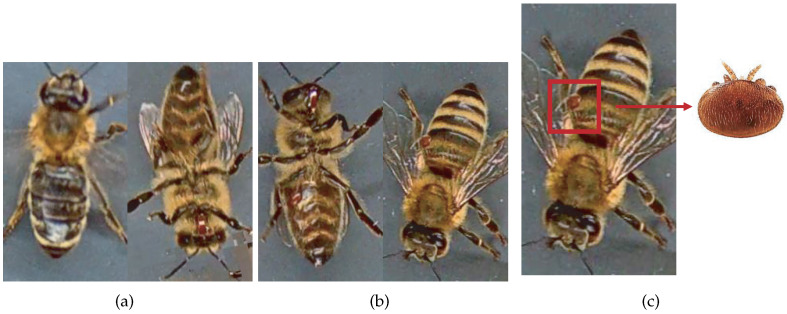
Some images from the VarroaDataset: (**a**) healthy bees, (**b**) bees with Varroa mites. (**c**) The Varroa mite has a reddish-brown coloration and usually hides under the tergite of the adult bee.

Most works compile and use databases unavailable to the public. However, VarroaDataset [17] is a public database available for Varroa detection. The dataset consists of 13,509 resolution images 160×280 px of healthy bees and bees infested with the Varroa destructor mite taken in a laboratory in a controlled environment and manually labeled. The predefined dataset is split into subfolders: train, test, and validation subsets, where the images of bees and parasites are different in the three sub-datasets: train, test, and validation. The configuration is not static due to the parasites’ active movements and the bee’s position change [17]. Table 1 lists the dataset’s statistics provided by the authors [17]. Figure 1 shows some images of the dataset where the classes are labeled healthy bees and infested bees. The presence of the Varroa mite can be seen marked with a bounding box with the reddish-brown coloration that characterizes it.

### 2.2. Implementation and Comparison of DeepLabV3 and YOLOv5 Models

Our study directly compared two leading techniques in detecting the Varroa destructor based on leading model architectures in image analysis: DeepLabV3 and YOLOv5. Both models excel in their respective areas of expertise: semantic segmentation and object detection. However, the authors have taken a novel approach using these techniques to classify bees directly.

DeepLabV3 [41] is a state-of-the-art convolutional neural network model explicitly designed for semantic segmentation tasks, classifying each pixel in an image into one of several predefined categories. The model is the third version of the DeepLab series, which has set a milestone in performance and precision in this type of task. Instead of using a standard convolutional network that processes the entire image simultaneously, DeepLabV3 uses a series of filters of different scales to capture details at varying levels of granularity. In addition, it includes an atrous spatial pyramid pooling (ASPP) module that improves segmentation at multiple scales. ASPP accurately captures detail at different scales using parallel convolutions with varying dilation rates, allowing the model to handle objects of various sizes. DeepLabV3 has proven highly effective and accurate in multiple semantic segmentation tasks, providing cutting-edge results on several standard metrics. The architecture is implemented uniquely to classify bees as ’healthy’ or ’infected’ based on the presence of the Varroa destructor parasite [17]. This approach is innovative as it allows us not only to segment the image but also to obtain a direct classification of the condition of the bees in the images.

On the other hand, YOLOv5 [42] is the fifth version of the ’you only look once’ (YOLO) series of models, designed for real-time object detection tasks. This convolutional neural network architecture has become popular due to its fast and accurate performance. The architecture evaluates the entire image in a single pass, unlike other approaches that analyze several parts of the image separately, which facilitates its high processing speed. The network is designed to simultaneously predict the bounding box coordinates and object class, allowing you to handle multiple objects of different categories in a single image. Compared to previous versions of YOLO, YOLOv5 features speed, accuracy, and functionality improvements, including support for large-scale detection, multi-label classification, and instance segmentation. These features have led YOLOv5 to be widely recognized as one of the best choices for real-time object detection tasks in various practical applications. YOLOv5 is used to identify and locate infected bees and the Varroa destructor parasite in images, not only detecting them as individual objects but also classifying them to determine the health status of the bee colony [16].

To compare these models, we followed a standardized approach using the same validation sets used in the references mentioned, allowing us to compare our results with those reported in those studies directly. We did not use data augmentation techniques, as none of the reference studies used them. This approach allowed us to be on a level playing field with the references mentioned and provides a fair comparison of the effectiveness of our implementation of the models.

### 2.3. Color Space

There are different ways to define the color space to detail the color in a standardized form. Depending on their application, they are expressed in three or four color components; the best known are RGB, HSV, and YCbCr. In the RGB model, each color appears in its primary spectral components of red (*R*), green (*G*), and blue (*B*). Generally, it is used in monitors, web pages, and digital photography [43]. This space is an additive color model, described by the value of each component as an integer in the range of 0 to 255. On the other hand, the HSV model provides an intuitive color space for natural human perception. It also includes information on tone or hue (*H*), as well as its saturation (*S*) and brightness (*V*), which is used in color palettes, television, and digital video. Furthermore, it is easy to convert from RGB space to HSV using the following expressions [44],

(1) $$\begin{array}{cc} H & =arccos\frac{1/2[(R-G)+(R-B)]}{\sqrt{\left[{\left(R-G\right)}^{2}+\left(R-B\right)\left(G-B\right)\right]}},\\ S & =1-3\frac{min(R,G,B)}{R+G+B},\\ V & =\frac{1}{3}\left(R+G+B\right). \end{array}$$

Digital video and image processing use the YCbCr model. This defines color in terms of luminance (*Y*), blue chrominance (*Cb*), and red chrominance (*Cr*). The transformation from the RGB color space to YCbCr is given by [44],

(2) $$\begin{array}{cc} Y= & 0.299R+0.587G+0.114B,\\ Cb= & B-Y,\\ Cr= & R-Y. \end{array}$$

The presented models represent the color depending on their application in such a way as to facilitate their implementation. The information of each channel is easily implemented using the multichannel moments to characterize the data with a small number of descriptors to report objects based on their shape and color. For this reason, exploring their behaviors with different color spaces is interesting.

### 2.4. Multichannel Legendre–Fourier Moments

Moments of an image and their invariants to geometric transformations have been widely used in computer vision [45] and pattern recognition [46] applications. They can describe objects with changes in scale, translation, noise, and various factors involved in image acquisition. Recently, multichannel moments have been used for RGB image analysis because they perform better than their predecessors, quaternion moments [19]. Multichannel moments are defined as moments where each channel belongs to an element of a color space of an image $f_{c}\left(r,\theta \right)$. The moments of each channel are given by,

(3) $$M_{n,m}^{c}=\int_{0}^{2\pi }\int_{0}^{1}f_{c}\left(r,\theta \right)L_{n}\left(r\right)e^{\left(-jm\theta \right)}rdrd\theta$$

where *n* is the radial order, *m* the angular order, $e^{\left(-jm\theta \right)}$ is the Fourier exponential, and $L_{n}\left(r\right)$ is the Legendre shifted orthogonal polynomial. The recurrence relation is given by [26],

(4) $$a_{n}L_{n}\left(r\right)=\left(2r-1\right)L_{n-1}\left(r\right)-a_{n-1}L_{n-2}\left(r\right),$$

where r∈[0,1] and the coefficient $a_{n}$ is calculated as follows,

(5) $$a_{n}=\frac{n}{\sqrt{4n^{2}-1}}.$$

The initial calculation of the zeroth and first orders is given by,

(6) $$\begin{array}{c} L_{0}\left(r\right)=1,\\ L_{1}\left(r\right)=\sqrt{3}\left(2r-1\right). \end{array}$$

On the other hand, the channel *c* can be defined by different color spaces, such as

(7) $$\begin{array}{c} c=\left\{R,G,B\right\},\\ c=\left\{H,S,V\right\},\\ c=\left\{Y,Cb,Cr\right\}. \end{array}$$

The multichannel moments configuration can be adapted to analyze images with different channels to extract color characteristics.

One of the most important properties of orthogonal moments defined on a unit disk is their rotation and scale-invariant representation. If we consider that an image $f_{c}\left(r,\theta -\gamma \right)$ is rotated γ degrees, then $M_{n,m}^{\left(\gamma \right)}$ are related to the Legendre–Fourier moments of the original image by

(8) $$M_{n,m}^{\left(\theta -\gamma \right)}=M_{n,m}^{p}\exp(-im\gamma ).$$

Therefore, when computing the modulus of the Legendre–Fourier moments,

(9) $$\left|M_{n,m}^{\left(\gamma \right)}\right|=\left|M_{n,m}^{p}\right|,$$

it is shown that circular moments are invariant to rotation. A key aspect to consider in image analysis using Legendre–Fourier moments is scale invariance. This concept refers to the ability to identify and analyze an object, in this case a bee, regardless of the size or resolution of the image. However, this invariance only holds if the images are cropped to contain only the bee. Suppose the images of the same bee are obtained from different distances, and an adequate clipping is not carried out. In that case, the Legendre–Fourier moments lose their invariance property before this scale. The change in the distance from which the image is taken generates a variation in the perceived size of the bee that cannot be adequately managed at this time. However, scale invariance is achieved by mapping the image to a disk drive, regardless of the image’s resolution. This mapping allows a normalizing of the representation of the object, allowing the Legendre–Fourier moments to effectively identify and analyze the object in question, despite the variations in size or resolution. Table 2 shows an example of rotation and scale invariance. Note that the values in the descriptors remain constant at different degradations.

## 3. Multichannel Legendre–Fourier Moments for the Varroa Detection

Moments being naturally invariant to rotation, scale, and noise-robust eliminate the need for data enlargement or data reduction preprocessing. However, the acquisition of the dataset images is not static due to the active movement of the bees and mites; for this reason, the images have different positions. The database contains images from the dorsal and ventral sides. Furthermore, both classes consider information about the color and shape of the bee’s legs, wings, eyes, and mouth. Table 3 shows examples of images according to this classification.

When evaluating different approaches to detect parasites in bees, we considered re-splitting the database to obtain four classes of 500 images each. The new sets respect the original labels of the database through a manual classification separating the images into four categories: bee dorsal side healthy, bee dorsal side with Varroa, bee ventral side healthy, and bee ventral side with Varroa.

**Figure 2:**
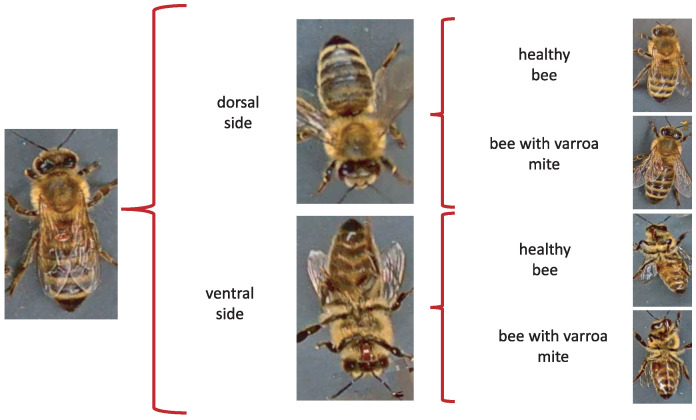
Varroa classification process.

The proposal consists of determining the initial orientation of the bee, such as the dorsal or ventral side, regardless of whether it is infested, to identify the presence of the Varroa mite later. Figure 2 shows the two stages of Varroa identification. First, the position is identified; subsequently, the Varroa mite is detected.

We use MATLAB R2021b (MathWorks, Natick, MA, USA) and the classification learner application in the model implementation with standard parameters. The computer hardware includes an Intel Core i7-9750H processor (Intel Corporation, Santa Clara, CA, USA) and 16 GB RAM. Additionally, a GeForce GTX 1050 graphics card (Nvidia Corporation, Santa Clara, CA, USA) is incorporated to achieve GPU acceleration, significantly enhancing the training processes of specific models.

The multichannel moments are calculated from the databases under normal conditions without preprocessing, with order five in the RGB, HSV, and YCbCr color models. Consequently, 75 descriptors are obtained, i.e., 25 per color channel. In this context, “color channel” refers to each component of the color model. For example, in the RGB model, R (red), G (green), and B (blue) are each a color channel. For each channel, 25 descriptors are calculated, leading to 75 descriptors. Therefore, each channel’s individuality in a color space dictates the number of descriptors, not the color space. Layering this information by channel allows for a more detailed and granular analysis, maximizing the amount of information that can be extracted from each image. The analysis considers the classification accuracy using a k-fold cross-validation strategy with k=10. Furthermore, the study implements a test suite of the VarroaDataset [17] ensemble to validate the robustness of our conclusions. This set was not used at any stage of training or cross-validation, thus ensuring its complete independence and allowing an unbiased assessment of the generalizability of our model. The final metrics we present in this paper, such as classification accuracy, are derived from this independent test suite. This approach provides a more rigorous and reliable validation of the effectiveness of our model, as it tests its ability to handle previously unseen data and ensures that our results are not biased by potential overfitting during the training phase.

The cubic support vector machine (SVM) algorithm is chosen to assess the proposal. The utilized images, as well as the multichannel Legendre–Fourier moment descriptors, are publicly available (https://www.kaggle.com/datasets/alicianoriega/varroadestructor-legendrefouriermoments (accessed on 9 July 2023)).

**Figure 3:**
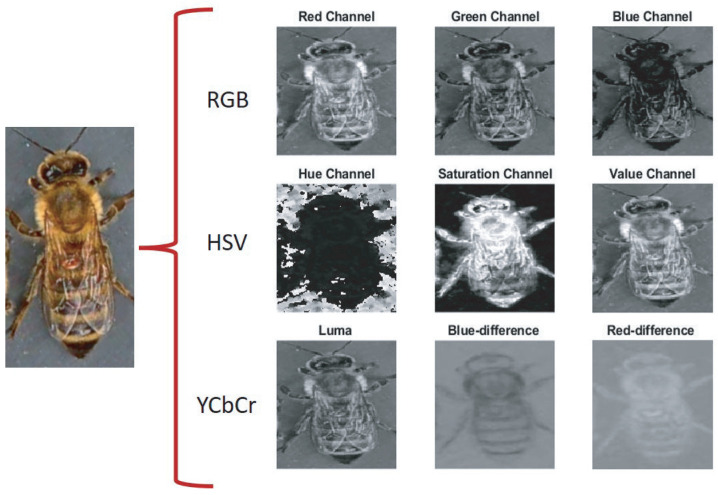
Classification with Legendre–Fourier moments using different color models.

**Figure 4:**
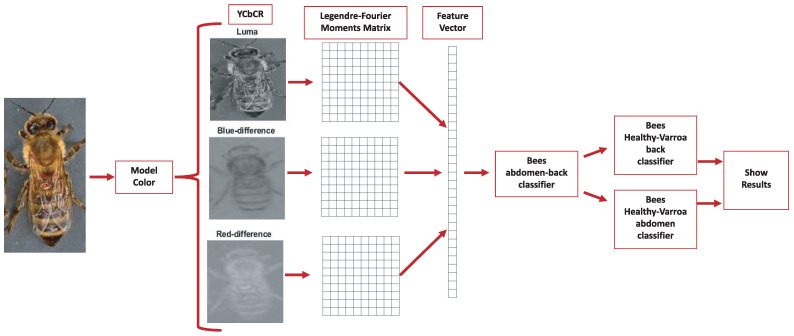
Diagram of the proposed classification process: classification with the YCbCr color space.

The proposal evaluates the performance of the descriptors in different color models to carry out the subclassification process. In the first stage, the features of the Legendre–Fourier moments are extracted for each channel of the selected color model. Figure 3 shows an example of the color models used and their respective channels. The descriptors are obtained to enter the first classifier to detect the bee’s orientation (dorsal side or ventral side). According to the output received, the second classifier identifies the presence of the Varroa mite. Figure 4 shows the proposed classification process for the YCbCr model.

The model performance evaluation metrics generally use the F1 score, conformed by the harmonic mean of precision and recall, the two most common metrics considering class imbalance. On the other hand, the confusion matrix or error matrix allows the visualization of the model’s performance. It makes it easy to check if the system is confusing two classes, i.e., mislabeling one class with another. From the confusion matrix, the accuracy and F1 score metrics are obtained. Table 4 shows the metrics used to evaluate the proposal.

## 4. Results

**Figure 5:**
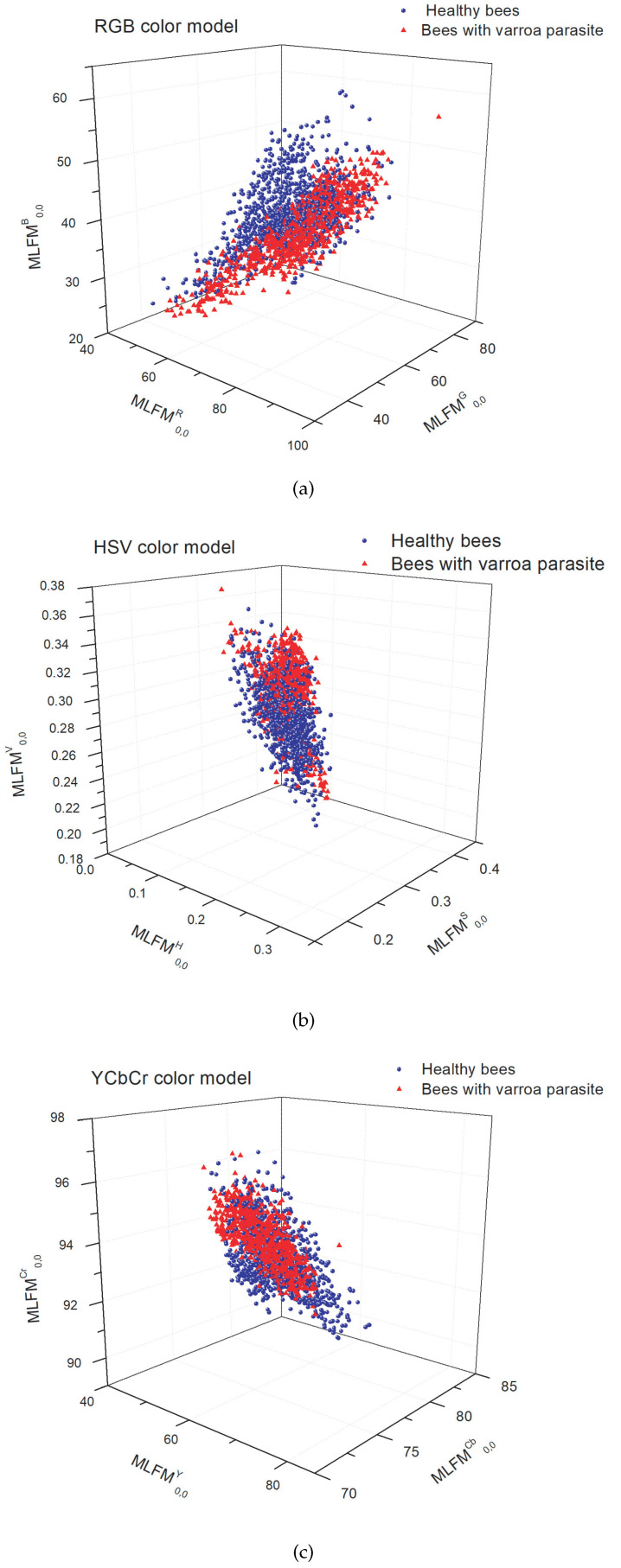
Scatter diagram of the classification: bees with healthy dorsal side and bees with healthy ventral side in the color models (**a**) RGB, (**b**) HSV, and (**c**) YCbCr.

The experiments explore the behavior of the MML-F as descriptors to classify the database with the subdivisions obtained. The first experiment organizes the database into two regular classes, healthy bees and bees infested with the Varroa parasite. Figure 5 shows the scatter plot of the classification with the three moment-type descriptors in the color models: RGB, HSV, and YCbCr. The image shows an intersection between classes that makes classification difficult.

Table 5 shows an accuracy of 91.86% was reached in the YCbCr color model. However, since they are living beings, it is difficult to capture the image in a single position of the bee [33]. Furthermore, other challenges are associated with distinguishing between the color of the mite and the bee’s eye. Therefore, it is crucial to consider the color information found on the bee’s dorsal and ventral sides [16]. Furthermore, it is important to note that mites often conceal themselves under adult bees’ sternites, adding a layer of complexity to their detection [6].

Using moment-type descriptors provides valuable information regarding shape and color characteristics. Therefore, the strategic subdivisions implemented in the database play a crucial role in distinguishing between various bee parts, such as the tongue, and the Varroa mite, even when they exhibit similar color patterns.

The following classification exercises consider the above issues to help determine the bee’s position and the Varroa parasite’s location on the host. The proposal uses the subdivision to perform the classification exercise. First, using 2000 images from the database, the classification determines the bee’s position, with the abdomen in a ventral or dorsal orientation. In the second part of the table, the accuracy in the classification achieved is 99.09% in the YCbCr color model.

The next step allows the determination of the presence of the Varroa parasite. Training is performed using data sets of bees, distinguishing between those with a healthy back and an infested back, those with a healthy ventral side, and those with Varroa on the ventral side. The accuracy increases for the first exercise and reaches 97.70% and 97.34%, respectively.

On the other hand, we use the SVM classifier with a cubic kernel because it has good results when used with moment descriptors in [47]. Furthermore, we perform the cross-validation with k=10 to validate the proposed model in the different color models. Table 5 shows the rates obtained with varying metrics in each classification exercise.

The experiments were performed with multichannel moments of higher orders; however, even when a higher order more accurately represents the images, it does not improve the results. In addition, the feature vector is more significant, which increases computation time and presents numerical instability in some cases. Lower-order multichannel moments provide both shape and color information; therefore, they are sufficient for higher classification accuracy.

Finally, all the exercises performed are used to train the classification models of the proposed algorithm for the RGB, HSV, and YCbCr color models to identify the space of color that provides more information for extracting characteristics from the Legendre–Fourier multichannel moments. The double classification carried out with the subdivision of the database allows the extraction of specific characteristics in training to increase the recognition rate of the parasite in bees.

The VarroaDataset has also been analyzed with different deep learning algorithms to identify Varroa in bees using semantic segmentation [17] and object detection models [16]. Table 6 shows the results obtained with different classification proposals and the proposed method.

## 5. Discussion

The results show a particular advantage with multichannel moments and the YCbCr color space over other color spaces and deep learning algorithms. The Varroa color group is more compact in YCbCr than in different color spaces. Furthermore, it has a minor overlap between the Varroa and honeybee data under various lighting conditions. The Y denotes the luminance component, and Cb and Cr represent the chrominance factors. Mainly, the Cr component highlights the characteristic red color of Varroa, thus facilitating the classification.

Furthermore, the difference between the classification results with YCbCr and RGB spaces is that the former represents color as brightness and two color difference signals. In contrast, the latter represents red, green, and blue colors. The color composition in the primary colors limits the classification of the Varroa. In HSV color space, it is separated by hue, saturation, and brightness, so it performs better than in RGB space. The tone component must highlight the Varroa, unlike the Cr of the YCbCr space.

On the other hand, the inherent characteristics of moments, such as their natural invariance to rotation and scale, allow classification with relatively small databases, unlike deep neural networks. Without a doubt, with more extensive databases and transfer of learning, deep neural networks can cover most inconveniences, such as the constant mobility of bees that causes Varroa to be confused with body parts; therefore, deep neural networks easily overcome the proposal. However, with the current dataset, the proposal presented is an excellent option for early detection of the Varroa destructor mite.

## 6. Conclusions

Currently, most of the applications in the identification of Varroa use segmentation techniques and convolutional neural networks, which present problems in differentiating the mite due to the information contained in the images of bees in training. On the other hand, the proposed algorithm has a high recognition rate by subdividing the database and using the color space to identify the Varroa mite more accurately. The accuracy in the classification achieved by the moment-type descriptors is possible due to the image characteristics used; the objects have a similar shape and color, differing by the characteristic color of the presence of the Varroa parasite. Low-order moment descriptors provide low-dimensional feature vectors and lower computation time without affecting their classification ability. In addition, they work with small databases and do not require data augmentation due to their invariance properties and noise robustness. The integration of the characteristics of the color spaces combined with the multichannel moments has also been explored.

Experimental results showed that this combination and subdivision of classes provide more accurate results than semantic segmentation [17] and object detection models [16]. The YCbCr color space presents a more significant advantage than other color spaces in combination with multichannel Legendre–Fourier moments because it highlights the features of the characteristic color of Varroa. There is a long way to go to improve the early detection of the Varroa destructor mite. However, the presented work lays the groundwork to explore other techniques for detecting Varroa by extracting features in different color spaces.

## Figures and Tables

**Table 1:** VarroaDataset statistics.

	Total	Train	Test	Val
Infested	3947	2554	942	451
Healthy	9562	5671	2466	1425

**Table 2:** Example of invariant Legendre–Fourier multichannel moments.

	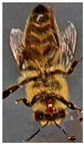	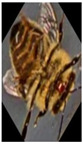	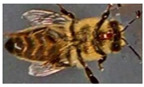	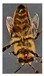	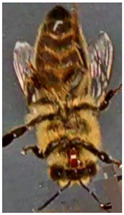
	Original	$\alpha \;=\;45^{\circ }$	$\alpha \;=\;90^{\circ }$	*k* = 0.5	*k* = 1.5
MLFMs					
$M_{0,0}^{B}$	53.023	53.023	53.023	53.023	53.023
$M_{0,1}^{B}$	0.628	0.628	0.628	0.628	0.628
$M_{1,2}^{B}$	2.080	2.080	2.080	2.080	2.080

**Table 3:** Subdivision made to the VarroaDataset database.

	Healthy	With Varroa Mite
Bee dorsal side		
	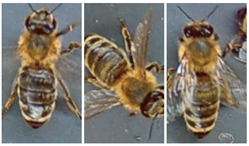	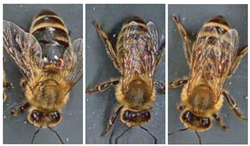
Bee ventral side		
	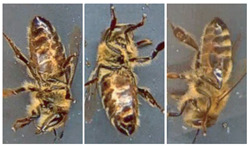	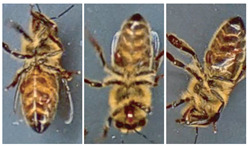

**Table 4:** Set of performance measures.

Measure	Formula
True positive rate (TPR)	TPR = TP/(FN + TP)
False positive rate (FPR)	FPR = FP/(TN + FP)
True negative rate (TNR)	TNR = TN/(TN + FP)
False negative rate (FNR)	FNR = FN/(FN + TP)
Accuracy	Accuracy = (TP + TN)/(TP + FP + FN + TN)
F1 score	F1 = (2 * TP)/(2 * TP + FP * FN)

**Table 5:** Set of performance measures.

	TPR	TNR	FPR	FNR	Accuracy	F1 Score
Healthy bees and bees						
with Varroa parasite						
RGB	89.5	87.6	12.4	10.6	88.5	88.4
HSV	92.4	89.4	10.6	7.7	90.9	90.8
YCbCr	91.7	92.0	8.0	8.3	91.9	91.9
VarroaDataset with subdivision						
Bees dorsal side and ventral side						
RGB	97.6	98.8	1.2	2.4	98.2	98.2
HSV	97.4	98.6	1.4	2.6	98.0	98.0
YCbCr	99.4	98.8	1.2	0.6	99.1	99.1
Healthy bees and Varroa-						
infested bee on dorsal side						
RGB	96.0	92.3	7.7	4.0	94.1	94.0
HSV	97.9	95.5	4.5	2.1	96.7	96.7
YCbCr	97.6	97.8	2.2	2.4	97.7	97.7
Healthy bees and Varroa-						
infested bee on ventral side						
RGB	95.7	94.7	5.3	4.3	95.2	95.2
HSV	97.4	96.2	3.8	2.6	96.8	96.8
YCbCr	99.6	95.4	4.6	41.8	97.4	97.3

**Table 6:** Classification results with VarroaDataset.

Model	Accuracy	F1 Score
DeepLabV3 [17]	90.8	95
YOLOv5 [16]	-	86.3
MLFM-RGB	94.1	93.9
MLFM-HSV	94.2	94.2
MLFM-YCbCr	96.7	96.6

## Data Availability

VarroaDataset [17] is a public database available for Varroa detection.

## Abbreviations

List of important symbols and abbreviations:

R	Red
G	Green
B	Blue
H	Hue
S	Saturation
V	Brightness
Y	Luminance
Cb	Chrominance
Cr	Red chrominance component
SVM	Support vector machine
TPR	True positive rate
FPR	False positive rate
TNR	True negative rate
FNR	False negative rate
